# Prepregnancy obesity and the risk of gestational diabetes mellitus

**DOI:** 10.1186/1471-2393-11-59

**Published:** 2011-08-10

**Authors:** Anussara Kongubol, Vorapong Phupong

**Affiliations:** 1 Department of Obstetrics and Gynecology, Faculty of Medicine, Chulalongkorn University, Rama IV Road, Pathumwan, Bangkok 10330, Thailand

**Keywords:** prepregnancy, obesity, gestational diabetes mellitus, preeclampsia, macrosomia

## Abstract

**Background:**

Prepregnancy obesity is associated with increased risk for adverse pregnancy outcome such as gestational diabetes mellitus (GDM), gestational hypertension, preeclampsia, fetal macrosomia and the need for cesarean delivery. The objectives of this study assessed whether Thai women classified as obese according to WHO's recommended body mass index (BMI) for Asians were at risk for developing gestational diabetes mellitus (GDM) and other complications such as preeclampsia, gestational hypertension and fetal macrosomia.

**Methods:**

Two hundred and forty women participated in this study and followed prospectively until delivery. Half of the women (n = 120) were obese (BMI ≥ 27.5 kg/m^2^) and the other half (n = 120) had normal weight (BMI > 18.5-23 kg/m^2^). Maternal demographic data, obstetric and neonatal outcomes from both groups were compared to each other. Relative risk and 95% confidence interval (CI) were calculated.

**Results:**

Compared to normal weight women, obese Thai women were not at increased risk for gestational diabetes mellitus (RR = 0.9 [95% CI 0.6-1.4]). Relative risk of preeclampsia and fetal macrosomia in obese women were 0.7 [95% CI 0.2-3.3] and 1.4 [95% CI 0.5-4.3], respectively. Relative risk of gestational hypertension in obese women was 12 [95% CI 1.6-90.8].

**Conclusion:**

When WHO's classification of obesity was used for Asian populations, prepregnancy obesity without metabolic problems did not increase the risk for GDM, preeclampsia and fetal macrosomia in Thai women. But, prepregnancy obesity continued to increase the risk for developing gestational hypertension.

## Background

Obesity is a major public health concern and its impact on pregnancy continues to grow in the world as well as in Thailand. The prevalence of obesity in Thailand has doubled in the past two decades. The prevalence of obesity in women with BMI ≥ 25 kg/m^2 ^increased from 23.2% in 1991 to 29.5% in 1997 and 34.3% in 2004 respectively [1]. Obesity contributes to significantly affect morbidity and mortality for several conditions such as heart disease, diabetes mellitus and hypertension.

There is an increase in number of interrelated adverse perinatal outcomes in obese women who become pregnant. Several studies have reported that maternal obesity is associated with increased risk for adverse pregnancy outcome such as gestational diabetes mellitus (GDM), gestational hypertension, preeclampsia, fetal macrosomia and the need for cesarean delivery [2-6]. Marked obesity is unequally hazardous to the pregnant woman and her fetus [7,8].

Most of these studies were performed in developed countries and its data may not applicable to the Asian population because Asians naturally have a lower body mass index (BMI), percentage of body fat, and health risks compared to Caucasians. As a result of this, WHO has adjusted its BMI recommendation for the Asian population [9]. Three specific factors led WHO to convene another expert consultation on BMI classifications. First, there was increasing evidence of the emerging high prevalence of type 2 diabetes and increased cardiovascular risk factors in parts of Asia where the average BMI is below the cut-off point of 25 kg/m^2 ^that defines overweight in the WHO classification. Second, there was increasing evidence that the associations between BMI, percentage of body fat, and body fat distribution differ across populations. Third, there had been previous attempts to interpret the WHO BMI cut-offs in Asian and Pacific populations, which contributed to the growing debates on whether there are possible needs for developing different BMI cut-off points for different ethnic groups [9,10]. Therefore, we conducted a hospital-based prospective study to investigate the effect of maternal prepregnancy obesity on pregnancy outcomes. The primary objective of this study assessed the risk for developing GDM in Thai women who were classified as obese according to WHO's recommended BMI for Asians. The secondary objective assessed other complications such as preeclampsia, gestational hypertension and fetal macrosomia.

## Methods

This study was approved by the Research Ethics Committee of the Faculty of Medicine, Chulalongkorn University. This study prospectively evaluates women with singleton pregnancies who attended antenatal clinic at King Chulalongkorn Memorial Hospital, Bangkok, Thailand before 28 weeks gestation between April 1, 2009 and April 30, 2010. Women between the age of 20-34 years attending antenatal clinics for the first time with no chronic medical illness, no history of GDM and preeclampsia, no family history of diabetes mellitus, and no history of fetal macrosomia (birth weight ≥ 4,000 g) were recruited into the study. Women with unknown body weight prior to pregnancy and had metabolic syndrome were excluded. Informed consent forms were signed by all eligible women prior to entering. The women were divided into 2 groups according to their prepregnancy BMI; women with normal BMI (18.5-23 kg/m^2^) became the controls whereas the obese women (BMI ≥ 27.5 kg/m^2^) joined the study group [9].

The sample size calculation was based on relative risks of primary and secondary outcomes obtained from our pilot study. We found that relative risks of preeclampsia gave the largest sample size. Thus, we needed 120 women in each group to detect a statistical difference (α = 0.05, β = 0.1).

A two-step diagnostic procedure using a 50-g glucose challenge test and a 100-g oral glucose tolerance test (OGTT) was used to diagnose GDM. 50-g glucose challenge test was performed at 24-28 weeks gestation. Plasma glucose ≥ 140 mg/dl was considered a positive result and was referred for a diagnostic 100-g oral glucose tolerance test (OGTT). GDM was defined as having > 2 plasma glucose values equal to or above the NDDG cut-off values (105, 190, 165, 145 mg/dL) [7]. Preeclampsia was defined as a new onset hypertension (BP ≥ 140/90 mmHg after 20 weeks gestation) and proteinuria ≥ 300 mg/24 hour or ≥ 1+ dipstick which regressed postpartum [7]. Gestational hypertension was defined as a new onset hypertension during pregnancy without any proteinuria which regressed postpartum [7]. The appropriate size of cuff was used to measure blood pressure. Fetal macrosomia was defined as fetal birth weight ≥ 4,000 gm [7].

Maternal age, parity, gestational age at first antenatal care and delivery, complications during pregnancy and delivery and neonatal outcomes were recorded.

Statistical analysis was performed by using SPSS software package version 12.0 (SPSS Inc, Chicago, IL, USA). Descriptive statistics was used and presented as mean, standard deviation, and frequency. Continuous variables were compared by student t test and Mann-Whitney U test. Chi square test (or Fisher exact test when appropriate) was used to compare categorical variables. Univariate and multivariate logistic regressions were used to explore the relationships between BMI and risk of outcomes; the results of the analysis were expressed as relative risk (RR) with 95% confidence interval (CI). A P-value < 0.05 was considered statistically significant.

## Results

A total of 240 women were enrolled in this study; 120 women had normal BMI and 120 women were obese. The demographic characteristics of women according to their BMI are summarized in Table 1. There were no significant differences for age, parity, and gestational age at delivery. Obese women weighted more, were taller and had a higher BMI compared to the women with normal BMI. But, the women with normal BMI gain more weight during pregnancy compared to the obese women.

**Table 1:** Demographic characteristics according to prepregnancy BMI

**Characteristic**s	Normal (N = 120)	Obese (N = 120)	P-value
**Age**	29.9 ± 3.5	28.2 ± 3.6	0.52
Parity*	1	1	0.098
**Weight (kg)**	51.8 ± 5.0	82.6 ± 11.6	< 0.001
**Height (cm)**	157.6 ± 5.4	159.0 ± 4.9	0.035
**BMI (kg/m^2^)**	20.8 ± 1.4	32.6 ± 3.8	< 0.001
**TWG (kg)**	16.0 ± 4.4	12.2 ± 5.7	< 0.001
**Gestational age at 1^st^ANC visit (weeks)**	12.4 ± 5.6	15.2 ± 7.2	0.001
**Gestational age at delivery (weeks)**	38.8 ± 1.1	38.8 ± 1.2	0.955
**No. of ANC visits (times)**	10.2 ± 2.3	9.3 ± 2.6	0.003

Data presented as mean ± SD or *median

TWG: total weight gain, ANC: antenatal care

Maternal outcomes between both groups are shown in Table 2. The rates of GDM, preeclampsia, preterm delivery, premature rupture of membranes (PROM), postpartum hemorrhage (PPH) and cesarean delivery were not different between both groups. Gestational hypertension occurred more frequently in the obese women compared to the controls (10% vs 0.9%, P = 0.004). Twenty-two (18.3%) cases of normal women and 23 (19.2%) cases of obese women need medication for control diabetes. All of these women have good control diabetes.

**Table 2:** Prepregnancy body mass index and maternal outcomes

	Normal (N = 120)	Obese (N = 120)	P-value
**GDM**			0.69
**- A1**	14 (11.7%)	10 (8.3%)	
**- A2**	22 (18.3%)	23 (19.2%)	
**Preeclampsia**	4 (3.5%)	3 (2.5%)	1.000
**Gestational hypertension**	1 (0.9%)	12 (10%)	0.004
**Preterm delivery**	4 (3.3%)	4 (3.3%)	1.000
**PROM**	7 (5.8%)	9 (7.5%)	0.605
**PPH**	1 (0.8%)	2 (1.7%)	1.000
**Cesarean delivery**	52 (45.6%)	64 (53.3%)	0.238
**Cesarean delivery due to CPD**	23 (44.2%)	36 (56.3%)	0.051

Data presented as n (%)

GDM: gestational diabetes mellitus, PROM: premature ruptures of membranes, PPH: postpartum hemorrhage, CPD: cephalopelvic disproportion

Neonatal outcomes between both groups are shown in Table 3. Birth weight was significantly higher in the obese women when compared to the controls. There were no significant differences between both groups for macrosomia, prematurity, Apgar scores at 1 and 5 minutes, and neonatal complications. There were no stillbirths in this study.

**Table 3:** Prepregnancy body mass index and neonatal outcomes

	Normal (N = 120)	Obese (N = 120)	P-value
**Birth weight (g)**	3178.9 ± 424.6	3322.5 ± 407.2	0.008
**Macrosomia (BW ≥ 4,000 g)**	5 (4.2%)	7 (5.8%)	0.554
**Prematurity**	4 (3.3%)	4 (3.3%)	1.000
**Apgar scores at 1 min**	8.8 ± 0.6	8.7 ± 0.7	0.55
**Apgar scores at 5 min**	9.9 ± 0.8	9.9 ± 1.1	0.798
**NICU admission rate**	0	0	NA
**Neonatal complications**			
**- Hypoglycemia**	0	0	NA
**- Infection**	0	1 (0.8%)	1.000
**- RDS**	0	2 (1.7%)	0.498
**- Hyperbilirubinemia**	23 (19.2%)	15 (12.5%)	0.157
**- Stillbirth**s	0	0	NA

Data presented as mean ± SD or n (%)

NICU: neonatal intensive care unit, RDS: respiratory distress syndrome

Relative risks of pregnancy outcomes are shown in Table 4. Only relative risk for gestational hypertension was considered significant (12, [95% CI 1.6-90.8]).

**Table 4:** Relative risks of pregnancy outcomes

	Relative risks	95%CI
**GDM**	0.9	0.6-1.4
**Preeclampsia**	0.7	0.2-3.3
**Gestational hypertension**	12	1.6-90.8
**Macrosomia**	1.4	0.5-4.3

GDM: gestational diabetes mellitus

## Discussion

This study evaluated the effect of obesity on pregnancy outcomes based on BMI for Asians. According to WHO'S BMI criteria, the range for normal BMI was 18.5-23 kg/m^2 ^whereas BMI ≥ 27.5 kg/m^2 ^was considered obese [9]. We did not find any association between obesity and gestational diabetes, preeclampsia, fetal macrosomia and the need for cesarean delivery. Our findings were similar to the results obtained from a South African study [11]. They also did not find any risk of gestational diabetes, preeclampsia, fetal macrosomia and the need for cesarean delivery in their obese women. They stated that complications associated gestational diabetes, preeclampsia, fetal macrosomia and the need for cesarean delivery were more commonly seen among morbidly obese women.

Furthermore, in present study, we found that gestational hypertension was significantly higher in obese Thai women. This finding corroborates data from another study conducted by Doherty et al [3]. However, we did not control for the risk factors of gestational hypertension. Thus, it may be difficult to interpret these results.

In contrast to these findings, results from previous retrospective studies [3-6,12], showed obese women having an increased risk for developing gestational diabetes, preeclampsia, fetal macrosomia and required cesarean delivery. The reason of this difference is due to the different BMI cut-off used to define obesity. In addition, obese women in present study controlled their diet during pregnancy. It has been shown that gestational weight gain during pregnancy is associated with adverse outcomes such as GDM, preeclampsia [13,14]. A study from Jain et al confirmed that the Institute of Medicine weight gain recommendation helped to achieve better pregnancy outcomes in obese and overweight women [15].

In another study conducted by Sahu et al [16], they showed that obesity was significantly associated with preeclampsia. But, we did not find the association between obesity and preeclampsia (RR 0.7, 95%CI 0.2-3.3) in present study.

As for neonatal outcomes, we did not find any difference between both groups for macrosomia, prematurity, or any other neonatal complications. This may be due to the fact that our obese women controlled their diet during pregnancy. This finding corroborates data from another study [17].

The strength of this study was its prospective nature. This allowed us to freely collect all data needed for the study and exclude confounding factors such as family history of diabetes, previous history of gestational diabetes. The other strength was that WHO's classification for obesity for Asians was never been used for evaluation the pregnancy outcomes. Unfortunately, one of the limitations of this study was its small case of some complications.

## Conclusions

When WHO's classification for obesity was used for Asians, prepregnancy obesity without metabolic problems did not increase risk for developing GDM, preeclampsia, fetal macrosomia nor require the need for a cesarean delivery. However, physicians should note that prepregnancy obesity can increases risk for developing gestational hypertension.

## Competing interests

The authors declare that they have no competing interests.

## Authors' contributions

AK and VP conceived, carried out experiments and analysed data. All authors were involved in writing the paper and had final approval of the submitted and published versions.

## Pre-publication history

The pre-publication history for this paper can be accessed here:

http://www.biomedcentral.com/1471-2393/11/59/prepub
